# Co-Producing Health Quality Management Improvements in Cardiovascular Disease, Diabetes, and Obesity Care in UAE: A Multi-Phase Study Protocol

**DOI:** 10.3390/ijerph23010006

**Published:** 2025-12-19

**Authors:** Nazik Nurelhuda, Md Hafizur Rahman, Zufishan Alam, Fadumo Noor

**Affiliations:** School of Health Sciences, Hamdan Bin Mohammed Smart University, Dubai P.O. Box 71400, United Arab Emirates; md.rahman@hbmsu.ac.ae (M.H.R.);

**Keywords:** health quality management, non-communicable disease, UAE, health care system, co-production, NCD, obesity, diabetes, cardiovascular diseases, knowledge translation

## Abstract

Cardiovascular disease (CVD), diabetes, and obesity pose major public health challenges in the United Arab Emirates (UAE), contributing substantially to morbidity, mortality, and healthcare expenditure. Despite progress in expanding access and service delivery, Health Quality Management (HQM) practices remain constrained. This study represents one of the first comprehensive, co-productive efforts to evaluate and strengthen HQM for CVD, diabetes and obesity in the UAE. Using a sequential, multi-phase design, it integrates evidence synthesis with the active engagement of interest groups to bridge gaps between research, policy, and practice. Phase 1 involves a scoping review to establish an evidence base on existing HQM practices and system-level challenges. Phase 2 conducts mapping and interviews with health professionals, policymakers, and patients to capture contextual insights. Phase 3 synthesizes findings to identify critical gaps, opportunities, and emerging research questions that can guide future inquiry. Phase 4 convenes consultative and consensus-building workshops to co-produce actionable recommendations and facilitate knowledge translation and exchange among health authorities, academic institutions, and other interest groups. Guided by the Institute of Medicine’s quality domains, the Donabedian model, and WHO quality indicators, this study situates HQM within the UAE’s ongoing shift toward value-based healthcare. The expected outcomes include the identification of key barriers to and facilitators of effective HQM, the formulation of context-specific recommendations to strengthen performance and coordination, production of knowledge translation outputs and the generation of new research priorities, thus contributing to achieving UAE Vision 2031 and global NCD targets.

## 1. Introduction

Noncommunicable diseases (NCDs) such as cardiovascular disease (CVD), diabetes, and obesity represent leading causes of morbidity and mortality worldwide, accounting for nearly three-quarters of all global deaths [1,2]. This burden is particularly pronounced in the Gulf region, where rapid urbanization and lifestyle transitions have contributed to some of the highest global rates of diabetes, obesity, and cardiovascular risk. In the United Arab Emirates (UAE), recent data indicate that diabetes affects approximately 17.3% of the population [3,4], obesity exceeds 35% among adults [5,6], and cardiovascular diseases remain the leading cause of death [2]. The rising prevalence of these conditions presents an urgent challenge to the country’s healthcare system and underscores the importance of strengthening quality management and preventive approaches within chronic disease care.

The UAE health sector is overseen by the Ministry of Health and Prevention (MOHAP) and by healthcare authorities such as the Department of Health–Abu Dhabi (DoH) and the Dubai Health Authority (DHA). While MOHAP is responsible for national health policy, regulation, and licensing, the Emirates Health Services (EHS), established in 2021, manages the operation of hospitals and primary healthcare centres across the northern emirates. This governance structure reflects the country’s effort to balance national coordination with emirate-level autonomy [7].

Over the past two decades, the UAE has undergone rapid transformation in its healthcare system, evolving into a mixed public–private model underpinned by universal access for citizens and mandatory insurance for residents [8]. The country’s unique demographic profile, with expatriates constituting nearly 80–90% of the total population, has been a defining factor shaping health service demand and policy priorities [9,10]. The population grew from 9.3 million in 2016 to approximately 11.0 million in 2024, largely driven by economic expansion and migration linked to service and construction sectors [11].

In response to this growth, the government launched system-wide reforms in the early 2000s to enhance efficiency, sustainability, and service quality [10]. These reforms introduced social health insurance, separated regulation from service delivery, and encouraged private sector participation to meet rising demand [12]. Today, the private sector accounts for a large majority of healthcare facilities and physicians [13]. Health financing reforms have been implemented across the UAE through emirate specific schemes. Prominent examples include Abu Dhabi’s Thiqa programme [14], which provides comprehensive coverage for Emiratis, and Dubai’s Saada scheme [15], which ensures inclusion of citizens not covered under other plans. In both emirates, employers are mandated to provide insurance for expatriate workers and their dependents, resulting in near-universal population coverage across the country [16].

The UAE has also strengthened its response to NCDs through the National Multisectoral Action Plan for the Prevention and Control of NCDs (2017–2021) and the formation of a National Multisectoral Committee under MOHAP to coordinate implementation [17].

The reforms have led to notable achievements in increasing the range of available services, improving service accessibility and attracting additional investments [18]. Despite this, several challenges persist. Increasing investments in healthcare have not been matched by proportional improvements in outcomes, while service overuse, fragmented regulation, and deficiencies in preventive care continue to strain system performance [10,13]. The impact of reforms on NCD outcomes remains unclear, underscoring the need for robust quality management frameworks to improve efficiency, coordination, and population health outcomes. Addressing these challenges requires the active involvement of multiple interest groups across the health system, including policymakers, regulators, healthcare providers, payers, and service users.

Health Quality Management (HQM) offers a useful framework for examining and strengthening health system performance. It refers to systematic governance and continuous improvement of health services to ensure they are effective, safe, people-centred, timely, equitable, integrated, and efficient, core dimensions identified by the World Health Organization (WHO), OECD, and World Bank as essential for achieving Universal Health Coverage [19]. Effective HQM integrates these principles into decision-making, performance monitoring, and accountability mechanisms to ensure that service expansion is matched by quality and equity in outcomes.

Building on this context, this proposal adopts a sequential four-phase methodology to study HQM practices for CVD, diabetes, and obesity, three major NCDs that together account for a significant burden of morbidity and mortality in the UAE. These conditions were selected because they represent both the leading causes of death and disability and a strategic research priority outlined in the UAE Ministry of Health and Prevention and Monash University’s National Health Research Strategy (2020), which identifies NCDs, particularly CVDs, diabetes, and obesity, as priority areas for advancing evidence-based research and improving national health outcomes [20]. This alignment ensures that this study directly supports national objectives and strategies for NCD management.

The overarching objective of this study is to comprehensively examine HQM practices for CVD, diabetes, and obesity in the UAE. The specific study objectives are to achieve the following:

1. Assess the current status of HQM practices in risk assessment, lifestyle interventions, medication management, and care coordination for CVD, diabetes, and obesity in UAE;
2. Explore the perspectives of key interest groups on existing HQM practices;
3. Identify gaps, barriers, and facilitators to effective HQM;
4. Co-produce evidence-informed recommendations with interest groups to strengthen HQM in line with national health priorities and the UAE Vision 2031.

This co-productive approach is expected to generate actionable insights to guide decision-makers, healthcare leaders, and practitioners in advancing an integrated, evidence-informed framework in the UAE [21].

## 2. Materials and Methods

### 2.1. Conceptual Framework for HQM

This study is grounded in a multidimensional conceptual framework that integrates the following three complementary perspectives to examine HQM within the UAE context.

- The Institute of Medicine (IOM) [22] quality domains namely safety, effectiveness, patient-centredness, timeliness, efficiency, and equity—define the essential attributes of high-quality care and establish the normative benchmarks for assessing service performance.
- The Donabedian model [23] evaluates quality across three interrelated dimensions: structure (resources, infrastructure, and governance), process (care delivery, provider–patient interactions, and coordination), and outcomes (health results, satisfaction, and system responsiveness).
- The World Health Organization (WHO) quality indicators [24] operationalize these principles into measurable components of system performance and accountability, linking national health service delivery to global quality standards.

This conceptual foundation guided the design, data collection, and analysis across all study phases, ensuring that quality management was explored from structural, procedural, and outcome perspectives relevant to the UAE’s health system context.

### 2.2. Sequential Multiphase Approach

**Figure 1:**
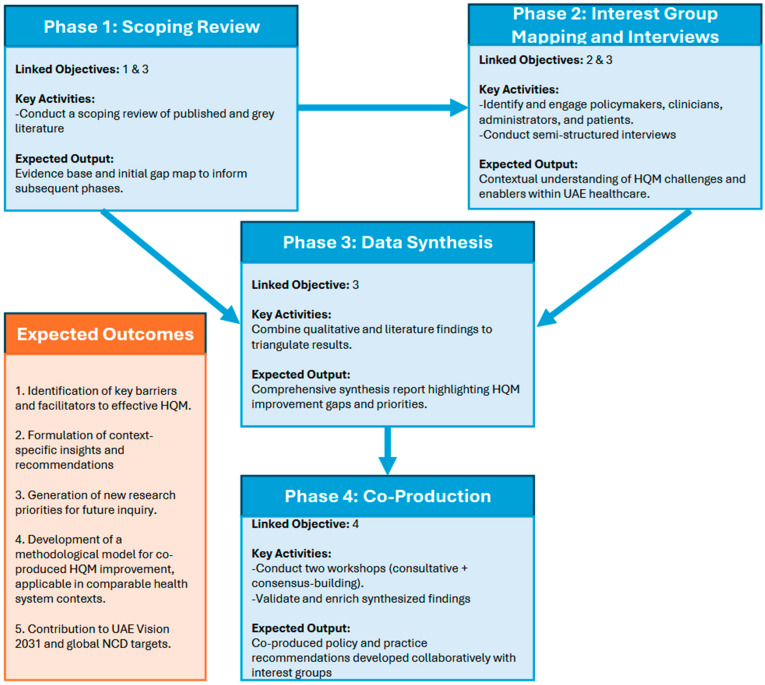
Sequential multi-phase co-production study framework and expected outcomes.

This study adopts a sequential four-phase methodology to evaluate and improve quality management practices for CVD, diabetes, and obesity in the UAE (Figure 1). Phase 1 involves a scoping review to establish a foundational understanding of existing HQM practices for the three respective NCDs within UAE, providing the evidence base for Phase 2, where interest group mapping and interviews will gather practical insights from key individuals and organizations. In Phase 3, the findings from the first two phases will be synthesized to identify critical gaps and opportunities. Finally, Phase 4 will engage interest groups in consultative workshops to validate results and co-produce actionable recommendations. This logical sequence ensures a comprehensive and informed approach, progressing from evidence gathering to interest group engagement and joint solution development, over a 12-month timeframe.

#### 2.2.1. Phase 1: Scoping Review

To assess the current status of quality management practices and examine gaps (specific objectives 1 and 3), a comprehensive scoping review of the peer reviewed research and grey literature will be conducted. The revised Arksey and O’Malley methodological framework [25] will guide our scoping review process, in accordance with the Preferred Reporting Items for Systematic reviews and Meta-Analyses extension for Scoping Reviews (PRISMA-ScR) guidelines [26].

Search strategy: The search strategy will utilize a combination of controlled vocabulary (e.g., Medical Subject Headings (MeSH) and free-text terms related to HQM, CVD, diabetes and obesity; and the UAE. Boolean operators (AND, OR) will be used to combine search terms effectively).
Search Concept: Health quality management, CVD, diabetes, obesity, UAE.
Search Words: Quality management, quality improvement, healthcare quality, cardiovascular disease, heart disease, diabetes mellitus, obesity, overweight, UAE, United Arab Emirates.
Databases: PubMed/MEDLINE, Scopus, and ProQuest.

Grey literature sources such as Google Scholar, institutional repositories, and relevant governmental websites will also be searched for guidelines, policy documents, recommendations, and reports, relevant to quality management of the three NCDs.

Time Period: No time restriction will be applied to capture a comprehensive range of literature. However, studies published within the last 10 years will be given priority to ensure relevance and currency.
Languages: English language articles will be included due to the predominant language of scientific literature. However, efforts will be made to include relevant grey literature published in Arabic by utilizing translation services if necessary.
Records Management and Screening: The retrieved records will be imported into the reference management software, Covidence [27]. The records will then be screened at title/abstract and full text levels against the eligibility criteria by two independent reviewers. To establish and document level of agreement between the reviewers, inter-rater reliability will be assessed using Cohen’s Kappa [28].
Scoping review analysis plan: Data from eligible sources will be charted and synthesized using a narrative and thematic approach. Extracted information will first be examined descriptively to identify patterns across study characteristics, disease focus, population groups, regional distribution, management strategy types and reported outcomes. Since interventional studies provide tangible evidence of quality improvement efforts and outcomes, subset analysis of studies falling under interventional design will be conducted. This will be complemented by guideline and recommendation documents from grey literature to contextualize the policy and practice landscape in the UAE. For the purpose of analysis, included records will be mapped out against the six domains of Institute of Medicine healthcare quality framework (safety, effectiveness, patient-centeredness, timeliness, efficiency, and equity) [22] and further categorized according to the Donabedian model to distinguish structural, process, and outcome-level quality measures [23]. Records will be assessed for report on relevant WHO quality-of-care indicators by quality dimension, in line with health quality domains, utilized in the multidimensional analysis study on quality of care and patient safety in European region [24]. The combined analysis will enable comparison across evidence streams, identification of key gaps and challenges, and synthesis of opportunities to strengthen quality management for priority non-communicable diseases in the UAE. Given the scoping nature of this review, critical appraisal of individual studies will not be conducted [29]. However, the methodological quality of included studies will be acknowledged and discussed within the narrative synthesis.

The findings from the scoping review will guide the development of the stakeholder interview guide in Phase 2, enabling targeted exploration of quality domains and issues uncovered in the literature. This approach will ensure that the interviews build upon the evidence from Phase 1, facilitating a comprehensive investigation of current practices, gaps, and opportunities for improvement in HQM within the UAE healthcare system.

#### 2.2.2. Phase 2: Interest Group Mapping and Interviews

Building on the findings from the scoping review, this phase aims to explore interest groups’ perspectives (Specific objective 2 and 3). The stakeholder mapping will identify key individuals and organizations involved in HQM, policy-making, and program implementation related to the targeted conditions, in addition to patient perspectives.

Mapping exercise: The WHO’s Stakeholder Mapping Guide, WHO Global Action Plan for the Prevention and Control of NCDs, the UAE’s National NCD Action plan [17,30,31] and the scoping review from phase 1 of this project will inform the interest group mapping process. The Kammi Schmeer stakeholder analysis guideline [32] will be employed to analyze interest groups based on their level of influence, interest, position, and relevance to NCD-related decision-making and service delivery, given its wide utilization in health policy and systems research. The prioritization and selection of interest groups for interviews will be conducted through accessing official websites of the identified potential interest groups, evaluating their organizational structures and activities, and selecting departments relevant to HQM.
Selection of study participants: This will follow a purposive and snowball sampling strategy to recruit approximately 25 participants, ensuring representation from each organization as well as identified individuals. This sample size is appropriate for qualitative research, allowing for data saturation while capturing diverse perspectives across stakeholder groups [33].
Data collection tools: Semi-structured interview guides (see Supplementary Materials) will be guided from IOM’s quality domains, the Donabedian model and WHO-clustered indicators for Quality of Care and Patient Safety. Probing questions will be informed from the findings of the scoping review. Distinct interview guides will be developed for each organization to ensure relevance to their specific roles and contexts (addressing Role and Contribution, Barriers and Gaps, Facilitators and Improvement Opportunities, and Collaboration and Partnerships) and patients (addressing Role and Engagement in Self-Management).
Data analysis plan: Qualitative data will be analyzed using Braun and Clarke’s (2006) six-phase thematic analysis framework, ref. [34] incorporating both inductive and deductive coding approaches through systematic familiarization, initial coding, theme identification, review, definition, and report production using qualitative data analysis tool, NVivo [35]. Data validation will be ensured through multiple strategies to enhance the rigor and credibility of findings. Two independent researchers will conduct parallel coding of the interview data, and inter-rater reliability will be assessed to evaluate consistency. Triangulation will be employed, including diverse stakeholder perspectives from different sectors (regulators, providers, academia, and civil society), to strengthen findings and enhance validity. Regular consensus discussions between the two coders will be conducted to resolve discrepancies, refine theme definitions, and ensure trustworthiness of the qualitative analysis throughout the coding process.

#### 2.2.3. Phase 3: Data Synthesis

To develop recommendations for improving HQM practices (Specific objective 3), data from the scoping review and interest group interviews will be integrated. Content analysis and thematic analysis will be performed using qualitative data analysis tools, such as NVivo [35], to identify gaps and opportunities for improvement. This synthesis will form the basis for informed recommendations.

#### 2.2.4. Phase 4: Co-Production Workshops

In the final phase 4, two workshops (Specific objective 4), one consultative and the second consensus building, will be conducted to validate and enrich the synthesized findings while developing collaboration among interest groups and co-producing insightful and actionable recommendations. This phase also aims to identify new questions, and research needs to enhance the practical relevance of the findings.

Ethical considerations: Ethical approval for this study was obtained from the Hamdan Bin Mohammed Smart University (HBMSU) Ethical Review Board, Dubai, United Arab Emirates. All study procedures will be conducted in accordance with the ethical standards of the institutional review board and the principles outlined in the Declaration of Helsinki.

## 3. Discussion

This study represents one of the first comprehensive, co-productive efforts to assess and strengthen quality management practices for CVD, diabetes, and obesity in the United Arab Emirates. Through a sequential, multi-phase design that integrates evidence synthesis with the active engagement of interest groups, it seeks to co-produce knowledge and bridge the gap between research, policy, and practice in noncommunicable disease (NCD) management [10,21]. This study responds to the national priority of advancing evidence-based approaches to prevention and chronic care, aligning with the UAE Vision 2031 and the WHO Global NCD Action Plan.

The scoping review will generate a detailed overview of current HQM practices and highlight system-level barriers across prevention, diagnosis, and care coordination. Applying established frameworks such as the Institute of Medicine’s quality domains [22] and the Donabedian model [23] provides a structured lens for benchmarking against international standards. Interest group interviews will add contextual depth by capturing real-world challenges and opportunities identified by policymakers, providers, and patients. Together, these components will generate a nuanced understanding of how HQM systems operate within the UAE’s health system and where they fall short in achieving equitable, efficient, and patient-centered care.

Building on the national shift toward value-based healthcare, this study also considers how HQM principles align with ongoing initiatives to link financing and performance metrics to health outcomes under the Vision 2031 agenda [19]. Embedding HQM within value-based frameworks ensures that efficiency gains are accompanied by measurable improvements in patient experience, safety, and population health.

Beyond documenting gaps, this research aims to identify leverage points for system improvement. The synthesis of findings across phases will help clarify where resources, governance, and data systems can be better aligned to support integrated NCD management. The consultative and consensus-building workshops will further ensure that recommendations are grounded in the realities of practice and co-developed with key actors, enhancing the likelihood of translation into policy and implementation [21]. These workshops represent a model of co-production, strengthening collaboration among health authorities, academic institutions, and interest groups.

The implications of this work may extend beyond the UAE context. By generating contextualized evidence and co-produced recommendations, this study contributes to the global discourse on quality improvement in similar countries undergoing rapid epidemiological and demographic transitions. The approach can serve as a model for other countries seeking to integrate prevention and chronic care into health quality frameworks, particularly where fragmented data systems and diverse service delivery models complicate policy coherence.

Certain limitations should be acknowledged. The availability of published data on HQM in the UAE may constrain the completeness of the scoping review. Likewise, interest group interviews depend on participants’ willingness and representation, which may introduce selection bias or limit generalizability. However, these limitations will be mitigated through triangulation across data sources, inclusion of diverse interest group perspectives, and validation through consensus workshops, enhancing the rigor and trustworthiness of findings.

Through this integrated and participatory approach, this study contributes to the UAE’s ongoing efforts to modernize its health system and improve population health outcomes. The expected outcomes, actionable recommendations, strengthened collaboration, and identification of future research priorities, can inform national strategies and guide resource allocation for long-term sustainability.

## 4. Conclusions

This sequential multi-phase study protocol outlines a framework for the co-production and assessment of HQM for cardiovascular disease, diabetes, and obesity in the UAE, offering a model for implementing co-produced approaches in comparable health system contexts. By integrating evidence synthesis with the engagement of key interest groups, it aims to generate actionable insights to strengthen NCD care. The findings are expected to contribute evidence to inform policy decisions, guide quality improvement implementation, and support more efficient and equitable service delivery. The expected outcomes include the identification of key barriers and facilitators to effective HQM, the formulation of context-specific recommendations, and the generation of new research priorities for future inquiry, thereby contributing to the achievement of UAE Vision 2031 and the broader goal of strengthening NCD management systems.

## Supplementary Materials

The following supporting information can be downloaded at: https://www.mdpi.com/article/10.3390/ijerph23010006/s1, Interview Guides—English Version.

## Abbreviations

The following abbreviations are used in this manuscript:

NCD	Non-communicable disease
CVD	Cardiovascular disease
CAP	Critical Appraisal Skills Programme
CINAHL	Cumulative Index to Nursing and Allied Health Literature
MeSH	Medical Subject Headings
