# Supplementary material for: Co-Producing Health Quality Management Improvements in Cardiovascular Disease, Diabetes, and Obesity Care in UAE: A Multi-Phase Study Protocol
Source: Int J Environ Res Public Health. 2025 Dec 19;23(1):6. doi: 10.3390/ijerph23010006 (PMC12840668; doi:10.3390/ijerph23010006)
Supplement: Supplementary file 1 [file ijerph-23-00006-s001.zip › ijerph-3897411-supplementary.pdf]

Interview Guides- English Version

<table>
<tr><td colspan="2">☐Health Regulator and Policy Makers
☐Healthcare Providers
☐Non-Health Agencies
☐Civil Societies and NGOs
☐Academia</td></tr>
<tr><td>Opening Statement-Introduction</td><td>Thank you for agreeing to participate in our research and this interview.
With your permission, I would like to audio record the interview because I don’t want to miss any of your comments. We would like to assure you that your or your organization’s identity will not be disclosed in the research and the collected information will be utilized only for the purpose of this study With your permission, may I turn on the recorder?

This research aims to explore interest groups' perspectives on quality in health management in Non-Communicable Disease (NCD) prevention and care practices particularly in the areas of diabetes, cardiovascular and obesity management to support policy development and practice improvement in the UAE.
We are interviewing you to better understand your role, challenges, gaps and facilitators in quality management in NCD care.
Quality in NCD Management refers to the delivery of effective, efficient, timely, safe, equitable, and patient-centered services across prevention, early detection, treatment, and long-term care. Through interest group mapping and in-depth interviews, we aim to capture the perspectives of both health and non-health actors, exploring roles and contributions, barriers and facilitators related to quality of prevention and management practices of NCDs, policy development and implementation, identify opportunities for improvement, and generate context-specific, equitable strategies for sustainable NCD prevention and management in the UAE.</td></tr>
<tr><td>Opening Question</td><td>Before starting with the main questions, can you please describe your role and your background?</td></tr>
<tr><td>Key Themes</td><td>Questions</td></tr>
<tr><td>Role and Contributions</td><td>1. Could you please describe your institutional role and contribution to quality management in NCD related services, particularly in the areas of cardiovascular disease, diabetes and obesity.
➢ Could you describe your organization's mission and primary activities?</td></tr>
</table>

	➢ How does your organization contribute to NCD (obesity, diabetes and CVDs) prevention, care or patient support or health promotion?<br>➢ What are your current priorities in NCD related health promotion, prevention and management/ research?<br>➢ How do you see your contribution aligning with the UAE National NCD Action Plan and other national strategies?<br>➢ How are your policies/ programs/ initiatives/ research influencing NCD outcomes in UAE?<br>➢ What policies/ services/programs/ research does your organization implement related to obesity, diabetes, or cardiovascular diseases? Can you discuss and share examples of these policies or initiatives?<br>➢ How are you developing and implementing these policies or programs?<br>➢ What specific populations or communities does your organization serve and how are they engaged in shaping NCD policies, programs, services/ research?
Perspectives on Quality Dimensions in Health Management	**Effectiveness** in health management refers to the degree to which health services, policies, and programs achieve their intended outcomes and contribute to improved health and well-being. It emphasizes doing the right things well by ensuring that interventions are guided by evidence-based practices in policy development, program design, and service provision (**All**)<br><br>2. So what are your perspectives on **effectiveness** of available policies or programs or initiatives related to NCD prevention and management?<br>➢ What are the main barriers or challenges?<br>➢ What are the gaps?<br>➢ What are the facilitators?<br>➢ How does your organization evaluate the effectiveness of NCD-related policies/programs/ Research?<br>➢ What indicators or outcomes do you use to measure success of your efforts?<br>➢ How do you integrate evaluation findings into future planning or improvement initiatives?<br>➢ What opportunities do you see for improvement? What would you like to change<br>➢ How could the connection between research, policy, and practice be strengthened to? (Academia only)

	**Efficiency** relates to optimal utilization and allocation of resources for NCD prevention and care (workforce, tests/ digital tools and telehealth) while maximizing the outcomes **(Regulator, provider, NGOs, non-health agencies)**<br><br>3. So what are your perspectives on **efficiency** in policy or program development/ service provision within your organization?<br>➢ What are the main barriers or challenges?<br>➢ What are the gaps?<br>➢ What are the facilitators?<br>➢ What tools or technologies have you used to help manage patient's condition? What worked well or not? (providers only)<br>➢ What opportunities do you see for improvement? What would you like to change<br><br>**Timeliness** refers to reducing waits and harmful delays for both those who receive and those who provide care, ensuring patients get the care they need when they need it, without unnecessary delays in diagnosis, treatment, or follow-up. **(Providers and Regulators)**<br><br>4. Please discuss how do you ensure **timely care or service provision?**<br>➢ What are the main barriers or challenges?<br>➢ What are the gaps?<br>➢ What are the facilitators?<br>➢ What policies, regulations, initiatives are available to ensure timely service provision?<br>➢ What opportunities do you see for improvement? What would you like to change<br><br>**Safety** in health means protecting patients/ people from harm while receiving care or services and ensuring that the processes, environments, and treatments are designed to minimize risks. It focuses on preventing errors such as medication mistakes, infections, surgical complications, or falls, and creating systems that make safe practices the norm **(Regulators, providers, non-health agencies, NGOs)**<br><br>5. Please discuss your perspectives on ensuring **safety** in NCD promotion, prevention or care delivery?<br>➢ What Protocols/ mechanisms exist to prevent medical errors or adverse events in NCD care<br>➢ What are the main barriers or challenges?

	➢ What are the gaps?<br>➢ What are the facilitators?<br>➢ What opportunities do you see for improvement? What would you like to change<br><br>**Equity** in health means ensuring that everyone has a fair and just opportunity to attain their highest level of health, regardless of factors such as age, gender, income, education, geography, or social background. It focuses on reducing avoidable differences in health outcomes between population groups by addressing barriers to access, affordability, and quality of care<br>**(Health regulators, providers and NGOs, Academia. Non-health agencies)**<br><br>6. Please discuss your perspectives/ role in ensuring **equity** in practices/ research related to NCD care?<br>➢ What are the main barriers or challenges?<br>➢ What are the gaps?<br>➢ What are the facilitators?<br>➢ What opportunities do you see for improvement? What would you like to change<br>➢ What policies/mechanisms/ initiative exist to ensure equitable access to care/ Ask about equity in NCD research.<br>➢ What policies/measures are in place to ensure reduction in out-of-pocket spending on NCDs (health regulators and providers only)<br><br>**Patient-Centeredness** ensures how patient needs and preferences are addressed, how patients are involved in the decision making, how care/ interventions are tailored to address patients' needs? (**Regulators, healthcare providers, NGOs, non-health agencies, Academia**)<br><br>7. What are your perspectives on addressing **patient-centeredness** in policy development, service and care provision?<br>➢ What are the barriers and challenges?<br>➢ Are there population groups you feel are underserved or overlooked in current NCD strategies or service provision / research?<br>➢ What are the gaps?<br>➢ What resources, support, or policy changes would be most helpful to close these gaps?

	➢ What are the facilitators?<br>➢ What opportunities do you see for improvement? What would you like to change
Collaboration and Partnerships	8. How do you/your organization currently collaborate with other interest groups in NCD prevention and management/ research?<br>➢ Please discuss any current collaborations/ projects<br>➢ What are challenges and gaps in collaboration?<br><br>9. How can collaboration between different interest groups e.g. government, providers, academia, civil society, and communities be strengthened?
Concluding Statement	10. Is there anything else you would like to add or discuss about NCD prevention, management, or quality of care?<br><br>Thank you very much for your time and I am sure that your input into this research will add immense value to further improve the quality of NCD prevention and management in UAE

**Patients and community representatives' questions**	
Opening Statement	Thank you for agreeing to participate in our research and this interview.<br>This research aims to explore interest groups' perspectives on quality in health management in Non-Communicable Disease (NCD) prevention and care practices particularly in the areas of diabetes, cardiovascular and obesity management to support policy development and practice improvement in the UAE.<br>We are interviewing you to better understand your role, challenges, gaps and facilitators in quality management in NCD care.<br>**Quality in NCD Management** refers to the delivery of effective, efficient, timely, safe, equitable, and patient-centered services across prevention, early detection, treatment, and long-term care. Through interest group mapping and in-depth interviews, we aim to capture the perspectives of both health and non-health actors, as well as patient groups exploring roles and contributions, barriers and facilitators related to quality of prevention and management

	practices of NCDs, policy development and implementation, identify opportunities for improvement, and generate context-specific, equitable strategies for sustainable NCD prevention and management in the UAE.<br>So, with your permission, I would like to audio record the interview because I don't want to miss any of your comments. We would like to assure you that your identity will not be disclosed in the research and the collected information will be utilized only for the purpose of this study<br>With your permission, may I turn on the recorder?
Opening Question	Could you briefly introduce yourself? You may include anything you'd like to share about your background — such as your education, age, occupation, and family (for example, your parents, siblings, spouse or partner, and children). Who do you currently live with? Your medical condition?<br>Where do you receive your care from? Government/ private/ hospital/ clinic?
Role and Engagement in self-management	1. In your daily life, what do you do to manage your condition and look after your health?<br>➢ What are the major challenges you face in self-managing your health condition at home?<br>➢ Have you participated in any community services, patient groups, or activities (e.g., exercise classes, educational sessions) that help manage your condition?
Perspectives related to quality dimensions in NCD management	**Effectiveness** means providing care that works—using treatments and services that are proven to help patients, so they can achieve the best possible health outcomes<br><br>2. From your experience, how well do the treatments or services you receive help to effectively manage your condition?<br><br>➢ What are the main barriers or challenges?<br>➢ What are the gaps?<br>➢ What are the facilitators?<br>➢ What opportunities do you see for improvement? What would you like to change?<br><br>**Efficiency** means making the best use of available resources—like doctors, nurses, medical tests, digital tools, and telehealth services—to prevent and manage chronic diseases, while also getting the best possible health results for patients

	3. Please describe your experience in navigating healthcare services (appointments, referrals, coordination)?<br>➢ How do you feel about the way different providers/organizations (hospitals, family doctors, community services, social groups) work together to support your care?<br>➢ What tools or resources you use to better manage your health condition?<br>➢ Any challenges you faced related to use of technology? Please describe those challenges.<br>➢ Please describe any positive experience or points in navigating the healthcare services provided by your healthcare provider.<br>➢ What would you like to change about the care coordination between the healthcare providers?<br><br>**Safety** means making sure that the care you receive does not cause you harm—by giving the right treatment at the right time, in the right way<br><br>4. How do you feel about the way healthcare providers manage risks or prevent mistakes when treating your condition? How safe do you feel your care is?<br><br>➢ What are the main barriers or challenges?<br>➢ What are the gaps?<br>➢ What are the things that make your care safe?<br>➢ What opportunities do you see for improvement? What would you like to change?<br><br>**Timeliness** means getting the care you need without unnecessary delays—like seeing a doctor quickly when you are sick, receiving test results on time, or starting treatment as soon as it is needed<br><br>5. Please discuss your experience with waiting times for appointments, tests, or treatments with your healthcare provider? How has it affected your care seeking behavior or management of your condition?<br>➢ What are the main barriers or challenges?<br>➢ What are the gaps?<br>➢ What are the things that help you get your care on time?

	➢ What opportunities do you see for improvement? What would you like to change?<br><br>**Equity** and access mean that everyone should have a fair chance to get the healthcare they need—no matter their age, gender, income, education, or where they live. It's about making sure services are available, affordable, and easy to reach for all patients, including those who may need extra support<br><br>6. Please discuss how equitable your feels to you?<br><br>➢ What challenges and barriers do you face?<br>➢ Have you ever had trouble accessing medicines, tests, or services? How did cost, location, or other factors affect your ability to access care and manage your condition?<br>➢ How does your language, socio-economic status, literacy, nationality affected the care provision by your provider?<br>➢ what makes access to care easier and fairer?<br>➢ What opportunities do you see for improvement? What would you like to change?<br><br>**Patient-centeredness** means putting you at the heart of your care. It's about listening to your needs, respecting your choices, and making sure treatment plans fit your values, culture, and daily life—not just the disease<br><br>7. How well do your providers understand and listen to your needs/preferences and include you in decisions to manage your condition?<br>➢ How well are healthcare providers meeting your needs?<br>➢ What are the challenges and barriers or gaps you face in getting your needs addressed by your provider?<br>➢ What helps make you care patient-centered?<br>➢ What kinds of support have you received that made it easier to manage your condition (e.g., family, healthcare providers, community organizations)?<br>➢ Have you used any technologies (apps, portals, wearables) to help manage your condition? What worked well or not?

	➢ How were your challenges addressed by your healthcare provider?<br>➢ Have you received any training, courses, or tools to support self-management? How useful were they?<br>➢ What kind of additional support, services, or resources would help you better manage your health? Any emotional, financial, practical, informational support would make the difference in helping you live well with your condition?<br>➢ What opportunities do you see to improve the patient-centered care?<br>➢ What would you like to change?
Concluding Question	8. What do you think about the alternative/ traditional medicine?<br>➢ Why would/ wouldn't you manage your condition through alternative/ traditional medicine?<br>9. Is there anything else you would like to add about the management of your condition or the services you receive?
